# Behaviour of Passive Fire Protection K-Geopolymer under Successive Severe Fire Incidents

**DOI:** 10.3390/ma8095294

**Published:** 2015-09-11

**Authors:** Konstantinos Sakkas, Alexandros Sofianos, Pavlos Nomikos, Dimitrios Panias

**Affiliations:** 1 Laboratory of Tunnelling, School of Mining and Metallurgical Engineering, National Technical University of Athens, 9 Iroon Polytechneiou St, Zografou, Athens 15780, Greece; E-Mails: sofianos@metal.ntua.gr (A.S.); nomikos@metal.ntua.gr (P.N.); 2 Laboratory of Metallurgy, School of Mining and Metallurgical Engineering, National Technical University of Athens, 9 Iroon Polytechneiou St, Zografou, Athens 15780, Greece; E-Mail: panias@metal.ntua.gr

**Keywords:** passive fire protection, tunnel linings, geopolymer, successive fires

## Abstract

The performance of a fire resistant coating for tunnel passive fire protection under successive severe thermal loading is presented. The material falls under the class of potassium based geopolymers (K-geopolymer) and was prepared by mixing ferronickel (FeNi) slag, doped with pure alumina, with a highly alkaline potassium hydroxide aqueous phase. Its performance was assessed by subjecting a concrete slab with a five cm thick K-geopolymer coating layer into successive RijksWaterStaat (RWS) fire incidents. During the first test, the maximum measured temperature in the K-geopolymer/concrete interface was 250 °C, which is 130 °C lower than the RWS test requirement, while, during the second fire test, the maximum temperature was almost 370 °C, which is still lower than the RWS requirement proving the effectiveness of the material as a thermal barrier. In addition, the material retained its structural integrity, during and after the two tests, without showing any mechanical or thermal damages.

## 1. Introduction

A number of serious tunnel fire incidents have been reported worldwide that have led to injuries and life losses, heavy damage in the concrete lining, excess material damage, and significant time periods of tunnel restoration during which the tunnels were unavailable for traffic.

Fires in tunnels can seriously damage their concrete lining rendering it to collapse. The damage is caused particularly by the spontaneous release of great amounts of heat and aggressive fire gases, resulting to spalling of concrete. The spalling phenomena are expected at several temperatures depending on the strength of the concrete. It is generally accepted that concrete exposed at temperatures higher than 380 °C is considered as damaged and should be removed and repaired [1]. In addition to the damage caused by fire to concrete, special attention has to be paid to the damage caused to the structural steel rebars that normally reinforce the concrete structures since are considered to lose their strength at temperatures between 550 °C and 600 °C [1,2,3,4,5]. Therefore, steel and concrete are both fire sensitive construction elements requiring passive protection against fire in order to be capable of withstanding of fire for an appropriate period of time without loss of stability.

Passive fire protection methods are generally divided in two categories: external (insulation) and internal (concrete design). The former are more advantageous being applied in new as well as in existing tunnels and consist of the cladding of the concrete by a fire resistant material which creates a protective external insulation envelope. However, even this kind of protection necessitates renovation of the tunnel lining after the fire. Most of the passive fire protection materials either lose their structural integrity or undergo severe damage which necessitates replacement of them. As a result they offer protection to the tunnel in case of one only fire. Apart from that, most of them necessitate long period of time for restoration and as a result tunnels remain closed with obvious negative economic effects.

This work aims at evaluating the performance of a fire resistant geopolymer for external passive fire protection of concrete tunnels linings under two successive RijksWaterStaat (RWS) fire incidents. Following the European Federation of National Associations Representing producers and applicators of specialist building products for Concrete (EFNARC) guidelines [6], its efficiency was assessed by subjecting a concrete slab specimen, coated with five cm K-geopolymer layer, to two successive thermal loadings employing the RWS curve, which is the worst case fire scenario among all the standard fire load curves.

The RWS curve considers a 50 m^3^ petrol tanker fire with a fire load of 300 MW lasting up to 120 min. The initial rapid fire growth is simulated with temperature rise up to 1200 °C within the first 10 min. Then, the temperature increases slowly and reaches the highest achieved value of 1350 °C within the next 50 min, followed by a gradual drop in temperatures to 1200 °C in the next hour as the fuel load is burnt off. The RWS temperature-time curve is able to describe very severe fire incidents in tunnels that are accidentally created by large tanker vehicles transporting oil or petrol. The performance requirements for an efficient fire resistant material are that the temperature of the interface between the concrete and the fire protective lining should not exceed 380 °C and the temperature on the reinforcement should not exceed 250 °C. In this paper, the behavior of the K-geopolymer under two successive RWS fire incidents will be evaluated, in order to test the possibility of leaving the material after the first fire incident on a tunnel lining without any restoration.

Geopolymers is a new family of synthetic aluminosilicate materials formed by alkali activation of solid aluminosilicate raw materials [7]. The geopolymerization reaction is exothermic and takes place at atmospheric pressure and temperatures below 100 °C [8,9]. Under a complicated mechanism, this reaction results in the formation of durable and compact amorphous to semi-crystalline solid materials characterized by a specific three-dimensional polymeric structure. Geopolymers possess excellent physicochemical, thermal and mechanical properties, like micro- or nano- porosity, high mechanical strength, notable surface hardness, thermal stability, fire and chemical resistance [8,9,10]. Due to these properties, geopolymers are viewed as alternatives for construction materials with excellent mechanical and unique thermal properties.

## 2. Experimental

### 2.1. Raw Materials

The slag used in the present study was provided by the metallurgical plant of the Greek company LARCO GMMSA (Larymna, Greece) that treats laterites to produce ferronickel. The slag is generated during the reductive smelting of laterites in electric arc furnaces and is granulated using a flash water cooling process. For the synthesis of the K-geopolymer, an adequate quantity of granulated slag was grinded to 90 μm and the resulted powder had a mean particle size (d_50_) of 15.05 μm. As is shown in Table 1, the FeNi slag is a siliceous material very rich in iron oxides and rich in alumina. It also contains substantial amounts of trivalent chromium, magnesium as well as calcium oxides. Pure metallurgical alumina, with mean particle size (d_50_) of 88.61 μm and purity higher than 99.4%, was also used for the synthesis of the K-geopolymer. Finally, a strongly alkaline potassium hydroxide solution was also used. The solution was prepared by dissolving pellets (Merck, Darmstad, Germany, 99.5% purity) of anhydrous potassium hydroxide in deionized water.

**Table 1:** Chemical analysis of the FeNi-slag used.

Species	*w*/*w* (%)
SiO_2_	41.14
Al_2_O_3_	13.79
FeO	34.74
Cr_2_O_3_	5.41
MgO	3.59
CaO	0.71

### 2.2. Synthesis-Experimental Procedure

The synthesis of the K-geopolymer was based on a previous research [10]. The fire resistant K-geopolymer was prepared according to the following procedure. A homogeneous viscous paste with the *w*/*w* (%) composition shown in Table 2, was initially prepared by mixing mechanically the alumina doped FeNi slag with 5.85 M potassium hydroxide solution. Then, the paste was molded in appropriate open plastic (Ertacetal) molds and was cured at 70 °C for 48 h. After curing, the specimens were de-molded and the two fire tests were carried out after 28 days.

**Table 2:** Synthesis of the K-geopolymer material.

Material	*w*/*w* (%)
Slag	42.24
Al_2_O_3_	25.03
KOH	8.09
H_2_O	24.64

### 2.3. Analysis and Tests

The mechanical properties of the K-geopolymer that were studied in this work include uniaxial compressive strength and flexural strength. The mechanical properties were measured in triplicate and the described values comprise the mean values of the measurement. Compressive strength was measured according to ASTM C109 [11] using cubic specimens of 50 mm edge. The flexural strength was measured according to ASTM C348 [12] using prismatic beam specimens of 40 × 40 cm^2^ cross section and 160 mm length. The properties were measured at 28 days and three months after the curing period. The thermal conductivity of the material was measured according to ASTM C518 [13] standard test using a 5 cm × 15 cm × 2 cm specimen.

The passive fire protection test was performed in the laboratory by using a test furnace which was designed according to European Federation of National Associations representing producers and applicators of specialist building products for Concrete (EFNARC) guidelines [7]. The furnace has the ability to simulate the temperature-time curves employed in several international standards such as the RWS fire load curve. For this test a 15 cm × 15 cm × 15 cm specimen was prepared, consisting of five cm thick geopolymer material and 10 cm thick concrete slab. The latter was prepared by using CEM II cement type (>350 kg/m^3^) and coarse aggregates from crushed limestone with maximum particle size between 16 and 20 mm as well as a water to cement ratio less than 0.48 according to the EFNARC guidelines. The test was performed 28 days after the production of the specimens.

During the test the free surface of the geopolymer material was exposed to a heat flux simulating the RWS fire load curve. The temperature at the geopolymer/concrete interface was measured by using a “K”-type thermocouple, while the temperature of the back surface of the specimen was measured with a high performance infrared thermometer. Two tests were performed with the same specimen in two successive days, simulating both of them the RWS curve.

## 3. Results and Discussion

### 3.1. Properties

The compressive strength of the K-geopolymer increased from 6.2 MPa to 8.7 MPa within the first 28 days and reached 9.5 MPa at 90 days. The flexural strength was 1.1 MPa at the first day after the geopolymer production and 2.1 MPa at the first 28 days. From the 28 days up to 90 days the flexural strength remained almost constant achieving a flexural strength of 2.1 MPa at 90 days from the production of the material. The thermal conductivity of the K-geopolymer was measured to be 0.16 W/(m·K) at 300 K. The density of K-geopolymer is 1800 kg/m^3^.

### 3.2. Fire Tests

**Figure 1:**
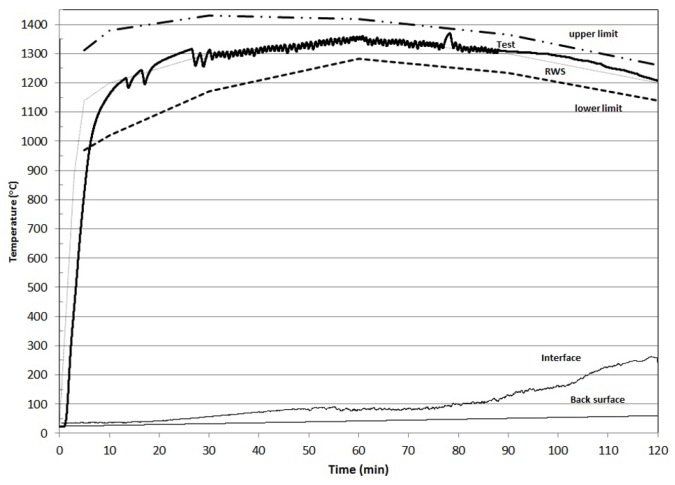
Performance of K-geopolymer at the first RijksWaterStaat (RWS) fire load curve.

The behavior of the K-geopolymer under the first fire loading test is shown in Figure 1, where the attained temperature at the concrete/geopolymer interface, the real furnace temperature simulating RWS fire load curve and the lower and upper limits of the performed RWS curve according to EFNARC guidelines are shown as a function of time.

**Figure 2:**
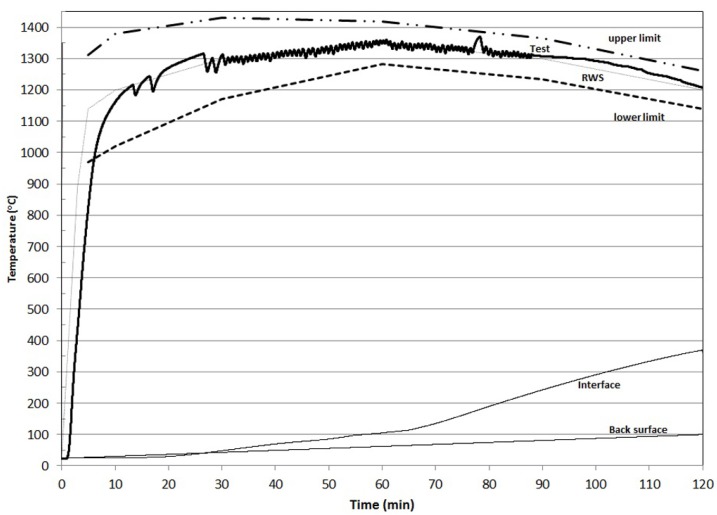
Performance of K-geopolymer at the second RWS fire load curve.

As it is observed, the temperature at the geopolymer/concrete interface was lower than 380 °C which is the requirement of the RWS curve during the whole duration of the fire test. The temperature reached 250 °C at the end of the test which is 130 °C lower than the performance requirements for an efficient fire resistant material set by the EFNARC for a passive fire protection test with the RWS fire loading curve. In addition, the interface temperature remained below 100 °C for almost the first 85 min of the fire test. At the first 25 min, where the temperature in the furnace were increased rapidly from the ambient temperature to 1250 °C, the interface temperature was remained at a temperature under 50 °C establishing a temperature gradient across the fire resistant geopolymer equal to 24 °C/mm. Then, the interface temperature started increasing reaching a plateau at 90–100 °C at about 55 min test duration. At this time, the furnace temperature was 1320 °C and the established temperature gradient across the geopolymer was even higher at 24.5 °C/mm. The interface temperature remained at about 100 °C for the next 25 min until the fire duration of 80 min. The temperature plateau at 100 °C is attributed to the removal of geopolymeric water through an endothermic water evaporation process consuming large amount of the incoming heat due to the large latent heat of water evaporation and keeping the interface temperature more or less constant at around 100 °C. From this point onwards, the temperature at the geopolymer/concrete interface started increasing while the furnace temperature decreased to 1200 °C. At the end of the fire test, the interface temperature was 250 °C and the temperature gradient was 19 °C/mm, which is the lowest value during the whole duration of the fire test. Finally, the temperature in the back surface of the concrete slab did not exceed 60 °C during the whole duration of the fire test, as is seen in Figure 1, which means that across the concrete slab the temperature was varied between 60 °C and 250 °C. After the end of the test, the specimen was cooled down at ambient temperature and during the next day the second fire test was performed under the RWS curve according to EFNARC specifications and guidelines. The results are shown in Figure 2.

As it is observed, the temperature at the geopolymer/concrete interface was slightly lower (=370 °C) than the RWS requirements of 380 °C during the whole duration of the fire test. In the second test, the interface temperature remained below 100 °C for 55 min instead of 85 min in the first fire test. At the first 20 min, where the temperature in the furnace was increased rapidly from the ambient temperature to 1200 °C, the interface temperature remained below 50 °C. The main difference with the first test is the time period during which the concrete/geopolymer interface temperature remains at the plateau of 100 °C. In the second test, the interface temperature does not reach a plateau at 100 °C because the geopolymeric water had been removed during the first test. Thus, the interface temperature constantly increased reaching the temperature of 370 °C at the end of the test. The temperature at the back surface of the concrete slab did not exceed 100 °C during the whole duration of the fire test, which means that across the concrete slab the temperature was varied in-between 90 °C and 370 °C.

**Figure 3:**
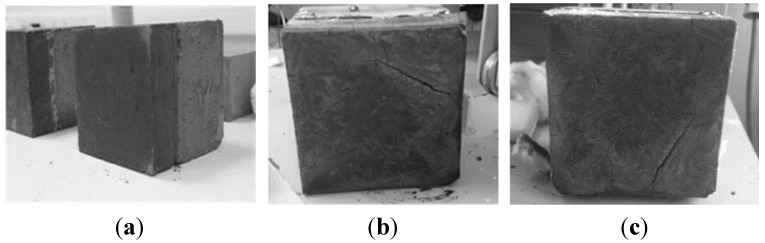
(**a**) Specimen before the fire tests. (**b**) Specimen after the first fire test. (**c**) Specimen after the second fire test.

In the photographs of Figure 3, the geopolymer before and after the fire tests may be observed. As it is seen the geopolymer superficial material after the end of both tests did not appear to have mechanical damage. It remained on top of the concrete slab without any change in its geometry. Even more, the surface of the geopolymer that was exposed during the fire tests directly at temperature as high as 1350 °C remained almost intact without cracks or other mechanical damages except some local, small in extent and not deep, superficial scaling. The concrete slab protected by the geopolymer did not present any spalling or other mechanical damage and remained as it was initially before the fire test.

The performance of the K-geopolymer under two successive RWS thermal loads was excellent. The material proved to have the ability to put an efficient heat flux barrier protecting the tunnel concrete lining as well as the steel reinforcement from the most severe fire incidents that may happen in underground constructions. In addition, the great macroscopic behaviour of the material after the fire tests without any mechanical damage or deformation proved that there is no need of renovation of the geopolymer material after the first fire test since it can withstand a second RWS curve according to EFNARC specifications and requirements. The material can remain on top of the concrete lining necessitating only minor interventions.

## 4. Conclusions

The fire resistant K-geopolymer from ferronickel slag that was studied in this paper demonstrated through a series of tests the following attributes:

- Sixty days of compressive strength reached the value of 9.2 MPa with a flexural strength of 2.1 MPa. The mechanical properties of geopolymer are comparable with the mechanical properties of the existing fire proofing materials and therefore the developed geopolymeric material has the potential to be used as a superficial material for passive fire protection of underground constructions. The thermal conductivity of the geopolymer is 0.16 W/(m·K) at 300 K, which is substantially lower from the thermal conductivities of the commonly used structural building materials.
- The behavior of the geopolymer during the fire tests under the most severe RWS fire load curve was excellent. The maximum temperature at the concrete/geopolymer interface was 250 °C after the first fire test and 370 °C after the second, which are both lower from the required temperature set by EFNARC for protection of tunnel concrete linings (≤380 °C). The K-geopolymer after the two fire tests did not appear mechanical damages as well as macroscopic deformations which prove that the material can remain on the tunnel after a fire without need of restoration. Additionally, in both tests, the K-geopolymer proved that can put an effective heat flux barrier offering very successful passive fire protection to the tunnel concrete linings as well as the steel reinforcement from the most severe fire incidents that can happen in underground constructions.

As a final conclusion, the designed K-geopolymer has been proved through small scale passive fire protection test to possess excellent fire resistant properties. This anticipated behavior should also be verified with a large scale as well as pilot tests before any application in practice takes place.
